# New and emerging concepts and therapies for the treatment of food allergy

**DOI:** 10.1093/immadv/ltac006

**Published:** 2022-02-04

**Authors:** David W Hwang, Cathryn R Nagler, Christina E Ciaccio

**Affiliations:** 1 Department of Medicine, The University of Chicago, Chicago, IL, USA; 2 Department of Pediatrics, The University of Chicago, Chicago, IL, USA; 3 Department of Pathology, The University of Chicago, Chicago, IL, USA; 4 School of Molecular Engineering, The University of Chicago, Chicago, IL, USA

**Keywords:** allergy and clinical immunology, immunotherapy, microbiome

## Abstract

Food allergy is an increasingly common disease that often starts in early childhood and lasts throughout life. Self-reported food allergy has risen at a rate of 1.2% per decade since 1988, and by 2018, the prevalence of food allergy in the United States was estimated to be 8% in children and 11% in adults.^-^ This prevalence has led to an economic burden of almost $25 billion annually. Despite these staggering statistics, as of the time of this writing, the Food and Drug Administration (FDA) has only approved one treatment for food allergy, which is limited to use in children with peanut allergy. Fortunately, a new horizon of therapeutic interventions, in all stages of development, lay ahead and hold promise for the near future.

Until recently, management of food allergy was limited to strict avoidance and preparation to rapidly treat a severe allergic reaction if accidental ingestion should occur. This management approach led to 20% of food-allergic children seeking care in an emergency department each year and 40% of food-allergic children reporting at least one severe allergic reaction leading to an emergency room visit in their lifetimes [1]. Further, avoidance can lead to significant anxiety in childhood and at times, social isolation at family gatherings, camps, celebrations, and sleepovers [2, 3]. For these reasons, current management options are suboptimal for many. Recently, several approaches have been under investigation for the treatment of food allergy that may soon lead to a paradigm shift in the management of this non-communicable chronic, disease (NCCD). In this report, we will review historical attempts at treating food allergy, discuss emerging goals of food allergy treatment and describe current innovative approaches to the treatment of food allergy in development.

Designing a therapy that can effectively treat food allergies is not a novel concept. The first known report of an attempt to treat food allergy was published in Lancet in 1908. In this report, an adolescent boy with egg “poisoning” who had experienced over 150 anaphylactic reactions to minimally cooked and baked eggs was co-administered calcium lactate with increasingly larger amounts of egg over 9 months until he was eating an egg a day [4]. As a result of this procedure, the boy was able to eat “anything”. The physician overseeing this early desensitization, Dr. Alfred Schofield, concluded that “some may think a great deal of trouble was taken to cure this idiosyncrasy, but when we remember that it was not connected with some rare food such as pineapple, which could easily be avoided, but with an article that enters into nearly all a schoolboy eats, and that his life had been more than once in danger from such food, it will be seen that the trouble taken was amply justified. The difference to the boy is, of course, enormous… .” Finding a treatment for food allergy was largely ignored until the early 1990s when the concept of utilizing immunotherapy was again explored. Early attempts at desensitization to peanuts, administered subcutaneously, resulted in high rates of systemic allergic reactions, including one death due to an erroneous dose [5, 6]. Although effective at increasing the threshold for reactivity, this form of immunotherapy failed to meet the suitable risk/benefit ratios stalling the pursuit of a food allergy therapeutic again.

In 2020, almost three decades after the first clinical trial for peanut immunotherapy began, the FDA approved a standardized peanut powder for use in OIT, as the first and only approved therapy for food allergy [7, 8]. This method of treatment for peanut allergy proved to be highly effective allowing nearly 70% of those on active therapy to tolerate 600 mg of peanut (or approximately two peanuts) without dose-limiting symptoms. Unfortunately, the side effect profile of this therapy as reported remained less than ideal with over 85% of subjects experiencing adverse events affecting the gastrointestinal tract and 81% experiencing adverse events affecting the respiratory tract. Systemic allergic reactions occurred in 14% of subjects [7]. As a result of this seminal work, interest in advancing the field of food allergy therapeutics has increased and is likely to accelerate in the coming decade. Many Phase 2 and 3 clinical trials are currently underway that may lead to FDA approval of therapeutic agents that have been shown in small, Phase 1 studies to have promising efficacy and side effect profiles.

## Definitions and goals of treatment

As the development of treatments for food allergy evolves so do the goals of treatment. Although finding a cure remains a priority for researchers, more immediate goals for treatment include desensitization and remission induction. “Desensitization” is a term that is used when food is given in increasingly larger doses to increase the threshold of reactivity. The threshold of reactivity, or amount of allergen needed to elicit a reaction, would then be sufficiently high to prevent a reaction upon exposure to a reasonable amount of allergen. The state of desensitization is dependent upon continued regular exposure to the food and quickly reverses if the food is withdrawn from the diet. “Tolerance induction” or “sustained unresponsiveness” are terms that are employed to imply that an individual will continue to tolerate a food for a period of time despite the withdrawal of the food from his or her diet on a regular basis [9].

These definitions have evolved in recent years as researchers acknowledge that goals of therapy are different for different individuals. For example, one may choose to undergo desensitization to a food or group of foods to raise his or her threshold of reactivity sufficiently high to make the odds of a severe or life-threatening reaction to an accidental ingestion exceedingly rare. This is increasingly termed “bite-safe.” Another individual, however, may only choose to undergo desensitization if it eventually results in his or her ability to freely eat the trigger food on a day-to-day basis. In contemporary parlance, immunotherapy and desensitization also do not necessarily imply the regular administration of a trigger food to alter the immune system, as studies are underway to determine if a monoclonal antibody or other biologic therapy alone may increase the threshold for reactivity or decrease the severity of a reaction to a food allergen. Similarly, “tolerance” and “sustained unresponsiveness” are commonly being replaced with the term “remission” which is more easily understandable to patients and acknowledges that the current state of tolerance (or the disease process) may change in the future.

## Immunotherapy

Allergen immunotherapy is currently the best-studied form of treatment for food allergies. Immunotherapy is a form of disease treatment in which a substance is used to modify the immune response. In the study of allergic disease, allergen immunotherapy implies that an allergen itself is being used to modify the immune response, specifically, increasing the threshold of reactivity [10, 11]. Four forms of allergen immunotherapy are currently being studied for use in food allergy, subcutaneous immunotherapy (SCIT), oral immunotherapy (OIT), sublingual immunotherapy (SLIT), and epicutaneous immunotherapy (EPIT). Although results are disparate, the basic principles of desensitization are similar regardless of the route of allergen immunotherapy. Typically, each allergen immunotherapy protocol starts with an updose phase (± – an initial dose-escalation day) in which the amount of allergen exposed and delivered to the immune system is slowly increased before reaching a maintenance phase. In the maintenance phase, a continued, regular exposure of the same allergen dose is delivered with the goal of continued immunomodulation [11]. As yet, the ideal length of therapy as well as the optimal maintenance dose for each route are unknown and may vary by individual. Reasonable goals of allergen immunotherapy may include continued regular exposure indefinitely, regular exposure at longer intervals indefinitely, or complete discontinuation with ad lib consumption of the allergen.

SCIT is a method of desensitization empirically used in the treatment of aeroallergen hypersensitivity since 1911. In the 1950s and 1960s, the first clinical trials were conducted to prove efficacy which paved the way for widespread use. In 1978, clinical trials were expanded to explore the use of SCIT in Hymenoptera hypersensitivity [12, 13]. For both indications, SCIT is performed using injections of dilute concentrations of allergen extracts which are increased weekly until a “maintenance dose” is achieved. When the maintenance dose is reached, injections are spaced to be given monthly for continued immunomodulation. Unfortunately, as discussed above, when this technique was tested in food allergy, systemic allergic reactions limited the advancement of the science [5, 6].

Due to the increasing incidence of food allergy, renewed interest in this mode of desensitization has led to significant progress in therapeutic development. In 2017, Vonk *et al*. demonstrated in a murine model that SCIT with peanut allergen decreased the signs of systemic allergic reactions upon peanut challenge. As seen clinically during desensitization, an increase in allergen-specific IgE was seen along with an increase in allergen-specific IgG1 and IgG2. Peanut SCIT also induced the cytokines IL-5 and IL-10 [14]. The same group later showed in a murine model that dietary supplementation with non-digestible short- and long-chain fructo-oligosaccharides decreased the incidence of anaphylactic reactions to SCIT to peanut; although the supplementation failed to improve the efficacy of this technique [15]. Simultaneous to this investigation, a chemically modified, aluminum hydroxide adsorbed, peanut extract was developed and is currently under investigation for the treatment of peanut allergy in humans. In 2017, preliminary data were published in an abstract form that indicated that early and late reactions in a group of 17 peanut allergy subjects “were generally of mild intensity and mainly consisted of redness and no wheal sizes exceeding 5 cm were recorded.” In addition, a trend toward reduced basophil activation was seen [16]. This led to an expanded Phase 1 clinical trial in the United States which recruited 42 peanut-allergic adults between 2016 and 2019 [17]. Published results were pending at the time of authorship.

The most extensively studied form of allergen immunotherapy to food is oral immunotherapy (OIT) with most of the work focused on milk, egg, and peanut. A comprehensive list of clinical trials involving OIT has been previously published [11]. OIT involves mixing increasing amounts of the trigger food with a semi-solid food, such as pudding, applesauce, or yogurt and orally administered daily. OIT is highly effective in inducing the ability to tolerate a small, accidental exposure to an allergen (make one bite-safe) but is associated with undesirable side effects. Early pivotal work demonstrated that 55% of children ages 5–11 could tolerate 5 g cumulative dosing of an egg after 10 months of OIT with a maintenance dose of 2 g of egg each day. After 22 months of OIT, 75% were able to tolerate a cumulative 5 g of an egg during the challenge demonstrating that OIT had a continued immunomodulatory effect during the maintenance phase of dosing [18]. More recently in the landmark paper discussed above, nearly 500 children ages 4–17 were enrolled to receive either peanut OIT or placebo. After 12 months of dosing (approximately 6 months of dose escalation followed by 6 months of maintenance therapy of 300 mg peanut protein), 67% of subjects were able to tolerate 600 mg of peanut protein without dose-limiting side effects [7]. In this cohort, 95% of participants experienced adverse events, including 60% graded as moderate and 5% graded as severe in the treatment group. Continued immunomodulation was demonstrated through a follow-up study of the same cohort that showed after 2 years, 80% (21/26) of participants could tolerate a 2000 mg dose (4043 mg cumulative dose) of peanut protein without dose-limiting side effects [19]. This work led to the first FDA approval of a treatment for peanut allergy in January 2020, a standardized product used for peanut OIT with both standardized egg and tree nut products under development [8, 20].

A potential advantage to OIT as compared to some forms of allergen immunotherapy is that dosing regimens can be easily adjusted in the offices of many practitioners to include more than one food (multi-food OIT) and personalize the different foods being administered during the desensitization protocol. This may be important as it is estimated that 30% of children are allergic to more than one food and these children experience a decreased quality of life compared to children with a single food allergy [21, 22]. In a small study comparing single food OIT to peanut and multi-food OIT to peanut and at least one other food, reaction rates did not differ between the two groups and dose escalations progressed at a similar rate. Efficacy was not determined in this early work, however [23]. In 2021, a Phase I/II study was launched to determine the safety and efficacy of a multi-food immunotherapy product in both children and adults with one or more allergies to the food allergens contained in the product [24]. If successful, this could be utilized as an OIT product in children and adults who are both mono- and poly-sensitized.

Due to the accessibility of food formulations suitable for OIT and the ability to individualize desensitization protocols, OIT has gained popularity in clinical practice in the last decade and accelerated the understanding of best practices [25]. As the food products used in OIT are frequently purchased over the counter, practitioners can make small adjustments to the amount of allergen given in a dose and the speed at which allergen exposure is increased with the goal of increasing tolerability of the desensitization process. In addition, multiple risk factors for systemic reactions during OIT have been identified and can, therefore, be avoided to improve the safety of the procedure. These risk factors include exercise immediately before or after dosing, hot showers, dosing on an empty stomach, fevers, sleep deprivation, menstruation, gastroenteritis, and other infections. Anticipatory guidance can not only help mitigate systemic reactions by avoiding risk factors while dosing or dose escalation but can also help anxiety and preparedness of mild reactions [26].

SLIT is an experimental form of immunotherapy that has shown benefit in food allergy with an improved side effect profile compared to other forms of allergen immunotherapy. In 2011, Kim *et al*. published the results of the study which enrolled 18 children ages 1–11 years who completed 12 months of peanut SLIT. In this study, a daily maintenance dose of 2 mg was achieved and the subjects in the active arm were able to tolerate 20 times more peanut on exit food challenge than those in the placebo arm. Like other studies of allergen immunotherapy, an initial increase in peanut-specific IgE was seen before a steady decrease. An increase in peanut-specific IgG4 was seen throughout. IL-5 levels decreased after the year, but no change was seen in IL-13 levels, the percent of T-regulatory cells, or the production of IL-10 and interferon-gamma [27]. In 2013, a multicenter study of subjects aged 12–37 with a history of peanut allergy showed 70% of individuals on SLIT for 44 weeks were either able to tolerate 5 g of peanut protein on oral food challenge or had at least a 10-fold increase in tolerated dose [28]. Further, 95% of doses given in this study were symptom-free if oral/pharyngeal symptoms were excluded. As has been shown in other forms of immunotherapy, SLIT demonstrated continued immunomodulatory effects throughout the maintenance phase of 5 years with 67% of participants who completed the therapy tolerating 750 mg of peanut protein on an exit food challenge. Because of the observed efficacy and safety of this form of immunotherapy, SLIT holds promise for future treatment protocols.

The final form of allergen immunotherapy discussed in this review is epicutaneous immunotherapy (EPIT). EPIT utilizes an adsorbed film or patch to deliver microgram doses of allergen to the epidermal layer of intact skin [29]. Similar to other forms of allergen immunotherapy, EPIT relies on slowly increasing doses of allergen achieved through varying times of skin contact with the patch. After a period wearing the patch, the goal of therapy is to initially increase the threshold for reactivity to make a child “bite-safe” then with continued exposure to induce tolerance or remission. In an animal model of ovalbumin (OVA) sensitized BALB/c mice, the tolerogenic role of allergen exposure on intact skin was demonstrated [30]. Dendritic cells were shown to internalize and transport OVA from the superficial layers of the stratum corneum to local lymph nodes leading to eventual upregulation of regulatory T-cells without systemic exposure. Advantages of this approach may include an improved side effect profile compared to OIT [7, 29].

The safety and tolerability of EPIT in peanut-allergic children and adults were demonstrated in a Phase 1 clinical trial leading to the eventual completion of the landmark PEPITES trial [29, 31–33]. In the PEPITES trial, 356 children ages 4–11 were enrolled and randomized to wear the peanut patch or sham patch for 12 months. The response was determined by either tolerance of 300 mg of peanut protein at exit food challenge for children who initially reacted to 10 mg or less of peanut protein or by tolerance of 1000 mg of peanut protein at exit food challenge for children who initially reacted to greater than 10 mg and less than 300 mg of peanut protein. The responder rate was significantly higher in children on EPIT compared to placebo and adherence was high. The overall incidence of adverse events was high, but reactions were primarily mild and limited to the patch site. After an additional 2 years of treatment with EPIT, this cohort demonstrated continued immunomodulation with 52% of subjects reaching an eliciting dose of 1000 mg or higher [34]. Unfortunately, the PEPITES trial failed to meet the prespecified confidence interval and is currently still in development before seeking FDA approval.

## Monoclonal antibody therapy

Monoclonal antibody therapy holds promise to be the next advancement in the treatment of food allergies. The goals of monoclonal antibodies, like other forms of food allergy treatment, are varied. To date, monoclonal antibodies have primarily been studied as an adjunctive therapy to OIT to decrease side effects and increase the likelihood of reaching a prespecified maintenance dose as discussed below. Interest has developed recently in understanding if using monoclonal antibodies as an adjunctive therapy could increase the likelihood of achieving sustained unresponsiveness or remission. In addition, studies are underway to determine whether a monoclonal antibody alone could sufficiently increase the threshold of reactivity to an allergen to make patients “bite-safe.” This idea is of particular interest as it would signal the advent of an allergen agnostic approach to food allergy treatment. Regardless of sensitization or even polysensitization, one injection could theoretically raise the threshold of reactivity for all allergens. Based on experience with monoclonal antibodies and asthma, a potential drawback of this approach is that it will require life-long administration to maintain clinical benefit. However, if this approach worked as a monotherapy and if the response to therapy is reliably rapid, the fourth goal of investigation may be to determine if administering a monoclonal antibody for short periods of time would sufficiently raise the threshold temporarily for “high risk” times in one’s life, such as while traveling, studying abroad, or during teen or college years. The success of such lines of investigation could lead to improved personalization of treatment plans.

The best-studied monoclonal antibody in the context of food allergy is omalizumab. Omalizumab is an anti-IgE molecule that gained its initial FDA approval for asthma in 2003 and has more recently also received approval for use in chronic idiopathic urticaria. Early work with omalizumab demonstrated its ability to decrease the side effects associated with OIT and the time to reach maintenance therapy [26, 35–37]. In addition, omalizumab has also been shown in small studies to increase the threshold of reactivity when used as monotherapy [38]. As this early work has demonstrated promising results, the omalizumab as monotherapy and as an adjunct therapy to multi-allergen oral immunotherapy in food-allergic children and adults (OUtMATCH) trial was recently launched in the United States. This trial aims to enroll 225 children and adults ages 2–55 years with an allergy to peanut and at least two other foods to test whether omalizumab alone or omalizumab plus multi-food OIT prevents allergic reactions to small amounts of the allergenic foods. The successful completion of this trial may signal the start of a paradigm shift in the treatment of food allergy favoring a personalized approach to management.

Dupilumab is a monoclonal antibody that acts as a dual inhibitor to IL-4 and IL-13 cytokine signaling and has been approved by the FDA since 2017 for the treatment of atopic dermatitis with subsequent indications for asthma and nasal polyps. In 2018, a case study reported a 30-year-old who tolerated two foods on oral food challenge to which she was previously reactive prior to starting treatment for atopic dermatitis with dupilumab [39]. Because of its effectiveness in other difficult-to-treat atopic conditions, dupilumab is now being formally explored for the treatment of food allergy in two unique studies [40]. In the first trial launched in 2019, dupilumab is being studied to determine its effectiveness as a monotherapy in children ages 6–17 with peanut allergies. In this study, the primary aim is to determine the proportion of children who tolerate peanut on food challenge after 24 weeks of treatment with dupilumab [41]. In a second study planned for enrollment, dupilumab is being studied to determine its effectiveness in increasing the number of children undergoing peanut OIT who tolerate an oral challenge to peanut after completion of updosing. This study includes several secondary aims, notably aims to determine if dupilumab improves the safety and tolerability of peanut OIT and aims to determine if long-term use of dupilumab after reaching maintenance provides added benefit compared to short term use just until maintenance dosing is achieved [42]. The comprehensive nature of these studies may help better predict how monoclonal antibodies are best utilized in the treatment of food allergies.

Ligelizumab is a high-affinity monoclonal anti-IgE antibody that has been found to have efficacy in early studies of chronic spontaneous urticaria and allergic asthma [43, 44]. Ligelizumab will soon be under investigation in a Phase 3 study in children and adults ages 6–55 years to determine if ligelizumab monotherapy is an effective treatment for peanut allergy by increasing the threshold of tolerance to 600 mg or greater of peanut protein. Secondary outcomes of this study include tolerance of 1000 mg or greater of peanut protein and 3000 mg or greater of peanut protein [45]. If successful, this study may bridge to future studies that treat this monoclonal antibody as allergen agnostic, successfully increasing tolerance to many or all foods in both monosensitized and poly-sensitized individuals.

In addition to the molecules discussed above, many other monoclonal antibodies in all stages of development are intriguing candidates for the treatment of food allergy. One example is lebrikizumab, a high-affinity anti-IL13 that has been shown to have efficacy in a phase 2b study of moderate to severe atopic dermatitis in adults and may be a candidate for future studies in food allergy [46]. Lirentelimab is a monoclonal antibody that targets a sialic acid-binding Ig-like lectin 8 (Siglec-8), an inhibitory receptor that blocks multiple allergic pathways. In a phase 2 study, lirentelimab improved symptoms in adults with eosinophilic gastritis or duodenitis [47]. As the number of monoclonal antibodies targeting the Th2 pathway continues to increase, the likelihood that one or more will prove efficacious in food allergy is promising.

## Microbiome-modulating agents

Food allergy is one of many non-communicable chronic diseases (NCCDs) that have undergone a marked generational increase throughout the industrialized world during the last 30 years. These NCCDs are linked by their association with changes in the commensal microbiome – the trillions of bacteria, viruses, bacteriophage, and fungi that colonize the skin and mucosal surfaces. Modern lifestyle practices including over-use of antibiotics, low-fiber diets, reduced infectious disease, Cesarean birth, formula feeding, and urban living have, collectively, reduced the diversity of microbes that populate 21st-century humans, depleting beneficial commensal taxa [48, 49]. Mouse model studies initially suggested that a bacteria-induced barrier protective response prevents allergic sensitization to food [50]. Subsequent work, using gnotobiotic models, showed that the healthy infant microbiota contains bacterial populations (not present in the cow’s milk allergic microbiota) that protect against an anaphylactic response to food [51]. This study provided proof of concept that feces contains microbial taxa (or metabolites) that can be identified and mined to treat food allergies. Moreover, two recent studies found striking taxonomic and metabolomic differences in fecal samples obtained from both healthy and food-allergic individuals [52, 53] Analysis of twin pairs, concordant or discordant for food allergy, revealed that the healthy twins were distinguished by their increased relative abundance of bacteria in the Clostridia class, the same taxa identified in the earlier infant study (and in the original mouse model), which they maintained into adulthood [50–52]. Clostridia are spore-forming bacteria, known for their ability to produce the short-chain fatty acid butyrate from the fermentation of dietary fiber. Butyrate, a major energy source for colonocytes is critical to the maintenance and function of the intestinal epithelial barrier [54, 55]. Infants whose fecal microbiomes display a reduced capacity to ferment butyrate are more susceptible to allergic sensitization [56]. Not surprisingly, then, there is great interest in trying to restore microbial “health”. An advantage of this approach is that it is “allergen-agnostic” since it treats microbial dysbiosis or dysfunction.

Many approaches are under study which range from transplantation of screened intact fecal material to the administration of defined bacterial consortia [57]. Fecal transplantation has shown efficacy for the treatment of *Clostridioides difficile* colitis and is currently in clinical trials for the treatment of food allergy [58, 59]. Much remains to be learned about the microbiome and, while fecal transplantation can be lifesaving in patients infected with *C. difficile*, the risk/benefit ratio is quite different in otherwise healthy children with food allergies. In this regard, transfer of defined, well-characterized consortia of bacteria (predominantly comprised of Clostridia) might be preferable; these trials are underway as well [60]. However, the experience thus far, across multiple NCCDs, has been that it is difficult to optimize long-term engraftment when working with highly oxygen-sensitive obligate anaerobes as live biotherapeutics. Promising results were seen in one study combining the readily available probiotic *Lactobacillus rhamnosus* CGMCC 1.3724. In this trial, 82% of participants who received the combination of a probiotic and peanut OIT achieved sustained unresponsiveness after 2–5 weeks of peanut consumption [61]. Unfortunately, this study lacked arms investigating the probiotic alone or peanut OIT alone; thus, the extent of possible synergism of the two therapies remains undefined. The future of microbiome-modulating therapeutics is likely to be the development of drugs based on microbial metabolites with a focus on restoring beneficial microbial function rather than replacing depleted bacterial taxa. Pairing microbiome-modulating drugs with monoclonal antibodies or immunotherapy may increase the likelihood of achieving remission.

With improvements in immunotherapy, effective biologics, and novel microbiome-based strategies, the next decade holds promise for many new treatment options for patients with food allergies.

## Data Availability

No new data were generated or analyzed in support of this manuscript.

## Abbreviations

EPIT: epicutaneous immunotherapy
NCCD: non-communicable chronic diseases
OIT: oral immunotherapy
OUtMATCH: omalizumab as monotherapy and as adjunct therapy to multi-allergen oral immunotherapy in food-allergic children and adults
OVA: ovalbumin
SCIT: subcutaneous immunotherapy
Siglec-8: sialic acid-binding Ig-like lectin 8
SLIT: sublingual immunotherapy
